# Prevalence and correlates of COVID-19 related anxiety among university students

**DOI:** 10.1192/j.eurpsy.2021.497

**Published:** 2021-08-13

**Authors:** E. Nikolaev

**Affiliations:** Social And Clinical Psychology, Ulianov Chuvash State University, Chebokasry, Russian Federation

**Keywords:** University Students, Anxiety, prevalence, COVID-19

## Abstract

**Introduction:**

COVID-19 is a disease with insufficiently studied diagnosis, therapy, and prevention that causes anxiety disorders in population.

**Objectives:**

To evaluate prevalence and correlates of COVID-19-related anxiety in university students during the period of their distant learning due to COVID-19 pandemic.

**Methods:**

The on-line survey of May 2020 covered 327 Russian university students aged 17-40. The questions concerned evaluation of threats, risks and acute problems faced by the students and their closest people in the situation of COVID-19 spread. We determined the anxiety level of the students by the degree of their concern about high risk of COVID-19 infection.

**Results:**

We established that 17.1% of the students had maximal level of COVID-19-related anxiety that correlated with older age (r=.13), better academic performance (r=.12), expectation of higher COVID-19-related threat to their life (r=.57), to the closest people’s health (r=.44), to the aged people’s lives (r=.16). It correlated with a more serious approach to evaluating the COVID-19-related situation and dangers in the world (r=.19), in the country (r=.24), and in the region of their residence (r=.37). Students with a high anxiety level often saw in pandemic a threat to their way of life (r=.12), material wellbeing (r=.12), and plans for the future (r=.11). They more strictly obeyed the restrictions (r=.13) and they did not exclude a recurrence of COVID-19 type pandemics in the future (r=.17).

**Conclusions:**

COVID-19 related anxiety is present in every sixth student and it correlates with older age and better academic performance. These students may have a high risk for depressive disorders.

